# Scrub typhus with multi-organ dysfunction syndrome and immune thrombocytopenia: a case report and review of the literature

**DOI:** 10.1186/s13256-019-2299-x

**Published:** 2019-12-07

**Authors:** Weijia Li, Lei Huang, Weixing Zhang

**Affiliations:** Department of Intensive Care Unit, Peking University Shenzhen Hospital, Shenzhen, 518036 People’s Republic of China

**Keywords:** Scrub typhus, Multi-organ dysfunction syndrome, Immune thrombocytopenia

## Abstract

**Background:**

Scrub typhus is an acute infectious zoonotic disease caused by *Orientia tsutsugamushi*. Multi-organ dysfunction secondary to scrub typhus is hard to diagnose and has a high mortality rate. Only one case of scrub typhus with multi-organ dysfunction syndrome and immune thrombocytopenia has been reported thus far. In this study, we report a second case of scrub typhus with multi-organ dysfunction syndrome and immune thrombocytopenia, and we summarize its diagnosis and treatment.

**Case presentation:**

A 43-year-old Han Chinese woman, a sanitation worker, was admitted to our hospital after 7 days of a skin infection and 5 days of a sore throat with fever and dizziness. A physical examination revealed the presence of an eschar on the right side of her neck. She had a history of insect bites during her sanitation work. A diagnostic evaluation identified scrub typhus as the primary illness, which was associated with multi-organ dysfunction syndrome and immune thrombocytopenia. She recovered completely after 15 days of treatment and extensive symptomatic supportive care.

**Conclusion:**

We report a second case of tsutsugamushi disease with multi-organ dysfunction syndrome and immune thrombocytopenia, which resolved after treatment and extensive care.

## Introduction

Scrub typhus is a rickettsial zoonotic disease transmitted by mites and caused by *Orientia tsutsugamushi* [1]. Scrub typhus is a severe public health problem that affects mainly Asia-Pacific areas; scrub typhus threatens one billion people and causes illness worldwide each year [2]. The original name of scrub typhus, which was given by Hashimoto in 1810, is “tsutsugamushi disease.” Although the median mortality rate of untreated patients is 6% and that of treated patients is 1.4%, the mortality rate of scrub typhus remains high and can reach up to 70% without proper treatment [3, 4]. The typical clinicopathologic symptoms of scrub typhus are abrupt high fever, severe headache, lymphadenopathy, generalized myalgia, eschar, and rash [5]. Accurate and effective approaches for the diagnosis of scrub typhus are lacking due to the absence of typical symptoms, which often leads to misdiagnosis and underdiagnosis. A pathologic characteristic of scrub typhus is disseminated vasculitis, which can cause damage to one or more organs, such as the liver, kidney, lung, brain, meninges, and skin [6]. The clinical manifestations of scrub typhus include acute febrile illness, malaise, high fever, headache, cough, disseminated intravascular coagulation (DIC), pulmonary edema, and hepatic dysfunction [5]. However, the most typical clinical manifestation of scrub typhus is a scab at the site of the bite of the mite. Worst of all, approximately one third of the cases may suffer from multiple organ dysfunction syndrome (MODS) during the course of the disease [7]. Thrombocytopenia is another critical clinical manifestation of scrub typhus [8, 9], and thrombocytopenia is one of the causes of MODS [10, 11].

Scrub typhus is rare and it is hard to diagnose and treat. As far as we know, there has been only one case of scrub typhus with thrombocytopenia and MODS reported [12]. In this study, we report a second case of scrub typhus with thrombocytopenia and MODS and discuss its diagnosis, pathological process, and treatment. Besides, it provides a reference for the diagnosis and treatment of complex tsutsugamushi disease.

## Case presentation

A 43-year-old Han Chinese woman, a sanitation worker, was admitted to our hospital after complaining of a skin infection for 7 days and sore throat with fever and dizziness for 5 days. After the onset, she took cephalosporin drugs (specific drug name and dose is unknown), the effect was not ideal. Two days before admission, she noticed a decrease in urine volume. In addition, she denied any medical history, except for gastric ulcer and angina pectoris, which were treated with unknown treatment protocols. She was married and living with her family, and she denied alcohol consumption and tobacco smoking.

**Fig. 1:**
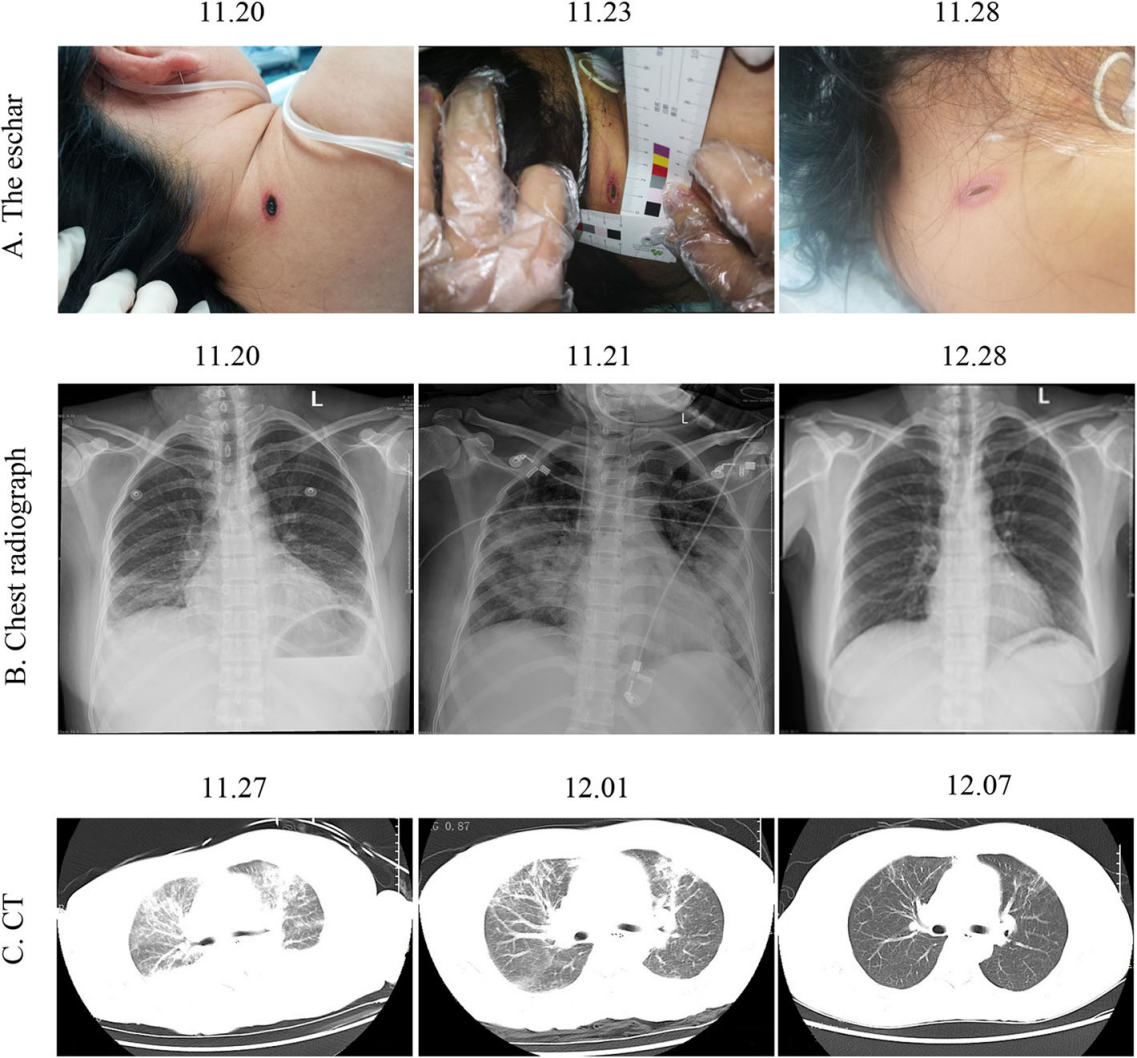
Changes in diagnostic examinations during treatment. **a** The change in the eschar during treatment. **b** The change in chest radiograph during treatment. **c** The change in computed tomography during treatment

At the time of admission, she was fully conscious; however, she had hypomimia. She was febrile (38 °C) with tachycardia (pulse 101/minute) and low blood pressure (85/41 mmHg). A physical examination revealed the presence of an eschar on the right side of her neck, which was approximately 2 cm × 1 cm in size (Fig. 1a). Babinski sign, Oppenheim sign, Gordon sign, and other pathological signs were negative. A lung examination by auscultation demonstrated fine, moist rales at the base of both lungs. After careful questioning and a review of her medical history, we noticed that she had received insect bites on her neck during her sanitation work.

Laboratory examinations upon admission revealed that hemoglobin was 113 g/L, her platelet count was 21 × 10^9^ platelets/L, and her white cell count was 10.09 × 10^9^ cells/L (8.88 × 10^9^ neutrophils/L, 0.74 × 10^9^ lymphocytes/L, and 0.46 × 10^9^ monocytes/L). Her coagulation function was significantly abnormal, where the prothrombin time was 24.80 (11.00–15.00 seconds), international normalized ratio was 2.25 (0.80–1.20), D-dimer was 12.64 (0–0.50 mg/L), and fibrin degradation products were 52.13 (0–5.00 mg/L), in addition to positive plasma protamine sulfate sub-coagulation results. Her renal function was also abnormal (serum creatinine 97 μmol/L). In addition, her liver enzymes were mildly elevated: serum alanine aminotransferase was 157 (9–66 U/L), lactate dehydrogenase was 1592 (313–618 U/L), total protein was 40.4 (63–82 g/L), albumin was 17.2 (35–50 g/L), serum total bilirubin was 59.7 (8.5–29.2 μmol/L), and serum conjugated bilirubin was 29.6 (0–5 μmol/L). There were also electrolyte disturbances, including hypokalemia (Na^+^ 127 mmol/L) and hyponatremia (K^+^ 3.40 mmol/L). Moreover, lactate was 3.77 (0.7–2.1 mmol/L), N-terminal pro-brain natriuretic peptide (NT-proBNP) was 555.4 (< 125 mmol/L), and interleukin 6 was 256.20 (< 7.0 pg/ml). Other laboratory tests did not initially show any obvious abnormalities. Chest radiographs and computed tomography (CT) indicated bilateral pulmonary exudation (Fig. 1b, c). Her Acute Physiology, Age, Chronic Health Evaluation II (APACHE II) score was 16, and the expected mortality rate was 8.7%. Based on her clinical history as well as the laboratory and imaging examinations, the following preliminary diagnoses were made: (1) tsutsugamushi disease, (2) septic shock, and (3) thrombocytopenia of unknown origin. Imipenem/cilastatin sodium (1 g every 8 hours) was used to treat the infection, and doxycycline (100 mg twice a day) was used to treat scrub typhus. In addition, she received symptomatic treatment, such as platelet supplementation, liver protection, and acid inhibition.

Eight hours after admission, she developed frequent cough and complained of dyspnea. Assisted ventilation support using a non-invasive ventilator was provided. However, there was no obvious improvement in her respiratory status. On the second day of admission, she was intubated with a synchronized intermittent mandatory ventilation mode. In addition, echocardiography indicated a depressed left ventricular ejection fraction of 62% and an increased NT-proBNP that reached 1701 mmol/L. Moreover, chest radiography showed that the bilateral pulmonary exudation had progressed with possible pulmonary edema. Due to her MODS (kidney, heart, lung, and brain), bedside continuous renal replacement therapy (CRRT) was given.

At that time, the Widal test, Weil-Felix test, and scrub typhus test were all negative. An human immunodeficiency virus (HIV) test, syphilis antibodies, and markers for viral hepatitis B and C were also negative. NT-proBNP increased to 7569 mmol/L at one time point and then returned to normal levels after symptomatic treatment. After 6 days, her vital signs were stable and the inflammatory indicators gradually decreased. Antibiotic treatment was changed to piperacillin tazobactam (4.5 g every 6 hours) and doxycycline (100 mg twice a day).

After 8 days, her platelet count increased to 200 × 109/L, and her liver and kidney functions and infection indexes returned to normal, except for BNP (753.9 mmol/L). Lung auscultation detected thick breath sounds in both lungs, with no obvious dry and wet rumble. The last chest radiograph and CT showed that the bilateral pulmonary exudation was significantly reduced (Fig. 1b, c). Our patient became conscious and was able to breathe autonomously with grade 4 muscle strength. In addition, the eschar had nearly cleared. As a result, she was extubated. However, the Widal test and the Weil-Felix test were still negative. Eleven days later, the antibiotic treatment was changed to moxifloxacin (0.4 g every day) and doxycycline 100 mg twice a day.

Fifteen days later, she recovered and was discharged. She did not experience any of the initial symptoms again, and all the indicators remained normal during the next 3 months.

## Discussion

In this case, our patient’s illness was caused by bug bites, which is a typical cause of tsutsugamushi disease. Because our patient did not pay enough attention to her condition, her condition worsened and finally developed into MODS with immune platelet decline. Fortunately, she received timely and accurate treatment; the prognosis of our patient is good. Compared with the existing literature reports, we draw the following summary: 1) all the causes were bug bites; and 2) the patients did not pay attention to their condition, resulting in the aggravation of their subsequent condition. However, what makes the case special is that our patient is a sanitation worker and is more likely to be bitten by bugs. Due to treatment delays, MODS occurs. However, because of the urgent condition of the patients, doctors rely on clinical experience to adopt a therapeutic diagnosis, which provides a new way for the diagnosis of complex tsutsugamushi disease.

The diagnosis of scrub typhus is a major challenge for clinicians, and as more organs are affected, the rate of misdiagnosis increases. One of the main reasons for the misdiagnosis of scrub typhus is that some clinicians are unaware of the complexity of the clinical manifestations of this disease [13, 14]. Eschar is the most important clinical manifestation for the diagnosis of scrub typhus [15], which usually forms around the swollen lymph nodes. In fact, eschars are painless, itchy, and are usually not easy to find; therefore, looking around the swollen lymph nodes usually helps to find eschars [16]. Skin ulcers on moist parts of the body, such as the perineum or armpits, are also an easily overlooked diagnostic clue for this disease [16, 17]. Nucleic acid detection, such as for HtrA (a 47-kDa periplasmic serine protease), the 56-kDa type-specific antigen rrs (16S rRNA), and groEL (the heat shock protein Hsp60), is accurate in the early phase of infection (until 10 days after the onset of fever), after which serology becomes superior for the diagnosis of scrub typhus [18]. In addition, recent studies have revealed that surface-enhanced Raman scattering-based lateral flow assay techniques are also rapid and accurate for the diagnosis of scrub typhus [19]. Loop-mediated isothermal amplification assays are also a diagnostic method for scrub typhus, but these assays are not widely used [20]. *O*. *tsutsugamushi* immunoglobulin G (IgG) in blood from patients is a biomarker of scrub typhus, and the gold standard method for the diagnosis of scrub typhus is an immunofluorescence assay (IFA). Recently, with high sensitivity, specificity, and reproducibility, improved anti-*Orientia* immunoglobulin M (IgM) and IgG-based rapid diagnostic tests and ELISAs have replaced subjective IFAs [19, 21–23]. Indirect IFAs have been the mainstay of scrub typhus diagnostics for decades, and we should pay attention to standardization, variable cutoff titers for endemic regions, and the high requirement for paired sera when using this assay [24]. In our case, no antibodies for scrub typhus were detected, and the feta test and the field test were also negative. Moreover, the laboratory indicators were atypical. The diagnosis of this disease was mainly based on the clinical manifestations (eschar, fever, and MODS) and diagnostic treatment (doxycycline treatment was effective).

There are some risk factors for determining the severity and prognosis of scrub typhus. Accumulating evidence has demonstrated that the frequency of eschars and rash in scrub typhus is closely associated with the infecting genotype [21]. In fact, 97% of all cases of eschar are caused by the Boryong genotype. On the other hand, virulent and severe diseases are usually caused by the Karp genotype [22]. Special attention should be paid to patients with scrub typhus with rapid progression that can lead to low body temperature, a rapid pulse rate, the presence of crepitation, a low percentage of lymphocytes, low serum albumin, elevated aspartate aminotransferase, elevated serum creatinine, and positive urine albumin [23].

Scrub typhus may cause thrombocytopenia and MODS. Previous studies have demonstrated that thrombotic microangiopathic syndrome is the mechanism of thrombocytopenia-associated MODS [25, 26]. A series of pathological changes such as thrombotic thrombocytopenic purpura (TTP), secondary thrombotic microangiopathy (TMA), and DIC may occur [25]. However, to the best of our knowledge, only one case of scrub typhus with thrombocytopenia and MODS has been reported thus far [12].

The early use of antibiotics is very important to avoid life-threatening scrub typhus [27], and a range of efficacious antibiotics are usually available for clinicians. In this regard, the effective antibiotics against scrub typhus are doxycycline, chloramphenicol, tetracycline, and azithromycin. Moreover, azithromycin may be more tolerable than doxycycline with the same effect [28]. On the other hand, randomized controlled trials suggested that clinicians should not use rifampicin as a first-line treatment option due to the low-certainty evidence and the risk of inducing resistance in undiagnosed tuberculosis [29].

## Conclusion

In this study, we reported a case of scrub typhus with thrombocytopenia and MODS, which is only the second case worldwide. In addition, the characteristics, diagnosis, pathological process, and treatment of this disease were described and discussed in relation to previously published cases in the literature. When people suffer from scrub typhus, we should pay attention to and prevent the occurrence of MODS and immune thrombocytopenia.

## Data Availability

All data generated or analyzed during this study are included in this published article.
